# Seminars may increase recruitment to randomised controlled trials: lessons learned from WISDOM

**DOI:** 10.1186/1745-6215-9-5

**Published:** 2008-01-29

**Authors:** Bronwen J Paine, Nigel P Stocks, Alastair H MacLennan

**Affiliations:** 1 Department of Obstetrics & Gynaecology, The University of Adelaide, Adelaide 5005, Australia; 2 Department of General Practice, The University of Adelaide, Adelaide 5005, Australia

## Abstract

**Background:**

Recruiting patients to large randomised controlled trials (RCTs) in the primary care setting can be challenging. Research teams need to identify and utilise strategies that both maximise the efficiency of recruitment and minimise the burden on general practitioners.

**Purpose:**

To describe our methods for identifying, approaching and recruiting female patients aged 50–69 years to a long-term double-blind RCT of hormone therapy (HT) – the Women's International Study of long Duration Oestrogen after Menopause (WISDOM). The effectiveness of conducting group seminars with patients prior to one-to-one screening is discussed.

**Methods:**

Female patients aged between 50 and 69 years were sent letters from participating general practitioners in Adelaide inviting them to participate in WISDOM and attend an initial seminar providing information about HT and the trial prior to a screening interview with a trial nurse. Recruitment rates for those who did or did not attend group seminars were compared.

**Results:**

Women who attended a group seminar conducted by the research team were twice as likely to attend an initial screening visit and enrol to participate in WISDOM than women who did not attend a seminar (p < 0.001). In addition, it was estimated that the time required to randomise a woman in the trial, and the number and duration of telephone calls to screen out uninterested women, was reduced for the seminar group.

**Conclusion:**

Conducting group seminars with potential participants may be a useful strategy for maximising recruitment from general practice, by increasing patient information and reducing a research team's workload.

**Trial registration:**

Current Controlled Trials ISRCTN63718836

## Background

Recruiting patients to large, population based randomised controlled trials (RCTs) can be challenging. Firstly, identification and screening of eligible patients in the chosen population is required, and this can be a lengthy process if the study in question has stringent selection criteria. Secondly, as with all research studies, time needs to be spent with each patient to ensure that they have a good understanding of the study's background, aims and procedures to enable them to make an informed choice about whether or not to participate. This process can be more involved when the study demands are substantial or the study requires a long-term commitment from its participants. In addition, if the study in question is a double-blind, placebo-controlled RCT, the specific principles unique to this type of study design need to be understood and accepted by participants. These principles include [1] clinical equipoise concerning the research question, reflecting collective professional uncertainty over a treatment's effectiveness, [2] randomisation to treatment or placebo and the inability to choose treatment and [3] the need for blinding to treatment by the patient, general practitioner and investigators.

Recruiting patients to RCTs in the primary care setting can be especially challenging. In this setting, research staff often rely on general practitioners (GPs) and practice nurses to identify and recruit eligible patients, and monitor patient health outcomes. However, the extent to which GPs and practice staff can identify and recruit patients for research is often limited due to time constraints and workloads. Thus, the research team often need to identify and utilise alternative strategies that both maximise the efficiency of recruitment and minimise the burden on GPs.

The purpose of this article is to describe our research team's methods of identifying, approaching and recruiting female patients aged 50–69 years for participation in a long-term double-blind RCT of hormone therapy (HT) – the Women's International Study of long Duration Oestrogen after Menopause (WISDOM) [1]. This was to have been the world's largest and longest randomised placebo controlled trial. In particular, the effectiveness of conducting seminars with patients in addition to one-to-one screening with a research nurse at the pre-recruitment stage is described.

WISDOM was an international trial conducted in the UK, Australia and New Zealand that assessed the long-term benefits and harms of HT use. WISDOM proposed to randomise 22,300 healthy postmenopausal women aged 50 to 69 years to 10 years treatment with oestrogen therapy, combined oestrogen plus progestogen therapy or placebo, and follow-up participants for an additional 5 years after treatment [1]. Four thousand of these participants were to be recruited in Australia. In October 2002, WISDOM was stopped following the early results from a similar long-term RCT of combined HT based in the USA – the Women's Health Initiative (WHI) [2,3]. The WHI investigators stopped the main arm of their trial – the combined oestrogen and progestogen therapy arm – when an increase in breast cancer rates (8 per 10,000 women years) seen at 5 years of HT use reached statistical significance, and an increased risk of stroke, thromboembolism, and heart disease was observed in participants [2]. At the time of WISDOM's closure, 5692 women had been randomised, and our Adelaide recruitment centre had screened a total of 840 women for the Australian arm.

## Methods

The original protocol for WISDOM recruitment in the UK involved a medical case note search of all women aged 50–69 in participating general practices [1]. The GP and trial nurse screened out those with obvious exclusion criteria and the remaining large majority were then informed about the trial by a postal letter, and then individually contacted by a nurse to discuss the trial and invited to a one-to-one information and screening interview for eligibility before obtaining informed consent to proceed to a pre-trial 'run-in' phase. Despite the recruitment of 384 general practices and a large recruitment team, recruitment was slow and fell behind target in the UK. Australia was next to commence recruitment and here it was decided to approach all women aged 50–69 years in each general practice by inviting them to group seminars about WISDOM, held in community venues near their practice. Ethical approval for WISDOM was given by the University of Adelaide Human Research Ethics Committee and the Royal Australian College of General Practitioners' National Research and Evaluation Ethics Committee.

### Invitation letter and written information

From May 2001 to July 2002, all female patients aged between 50 and 69 years were sent letters from participating GPs in Adelaide inviting them to participate in WISDOM and attend an initial seminar conducted by the research team. A reply slip, reply paid envelope (addressed to the research team) and WISDOM information sheet were enclosed with the invitation letter. The information sheet included detailed information about the trial's background, purpose and procedures, a list of broad inclusion and exclusion criteria, and a general explanation of RCT study design (including explanations of randomisation, use of placebos and blinding). The reply slip requested that women tick one of four responses as follows: [1] I am interested in participating in WISDOM and will attend the information evening, [2] I am interested in participating in WISDOM but unable to attend the seminar and/or would prefer a trial nurse to contact me by telephone, [3] I do not think I am eligible to participate in WISDOM based on the information provided in the enclosed information sheet, or [4] I am not interested in participating in WISDOM and want no further contact with the research team.

### Seminar

The lay seminars were conducted by the research team within 2 weeks of the initial invitation mail-out and were usually conducted in local community centre halls within close proximity of the woman's general practice. The seminar presentation, conducted by one or more of the local investigators, followed a standard Microsoft Powerpoint format and covered the following topics:

Part 1: Discussion of the currently known risks and benefits of HT

(1) The importance of evidence based medicine and the sources of information that provide health care professionals with knowledge of evidence based practice and the effectiveness of treatments.

(2) An overview of RCTs, with emphasis on this type of research method as the 'gold standard' for evaluating the effectiveness of health care treatments. The rationale behind placebos, randomisation, and blinding was also discussed.

(3) The currently known benefits and harms of HT for postmenopausal women, based on results from observational studies and short-term RCTs, were discussed. The statistical findings were reported, discussed and summarised in lay terms.

(4) The rationale for a long-term RCT of HT was explained.

Part 2: Explanation of WISDOM

(5) The background, purpose and procedures of the 15 year WISDOM trial was explained.

(6) The main inclusion and exclusion criteria of WISDOM, allowing women to self screen themselves.

(7) The potential benefits for taking part in WISDOM were discussed. This included individual benefits, such as health follow-up and monitoring, to global benefits, such as the contribution to knowledge about HT for future generations of women.

Part 3: Question time. This allowed general questions from the audience to be answered in front of the other women and accompanying partners, relatives or friends. Personal questions could also be answered privately at the end of the seminar.

At the end of the seminar, women were asked to complete a brief questionnaire asking whether they were interested in being telephoned by a WISDOM trial nurse to arrange a screening visit. The questionnaire also included a checklist of broad medical exclusions to confirm initial eligibility before screening.

### Telephone calls

Those who attended the seminar, and were still interested and eligible based on the post-seminar questionnaire, were telephoned by a trial nurse within one week of the seminar and invited to attend an initial screening interview. Women who did not attend the seminar but who gave a positive response of interest on their initial reply slip were also telephoned by the research nurse. Since these women had received only written information about WISDOM, the nurse also explained to them the main purpose and procedures of the trial, and confirmed their interest and initial eligibility before inviting them to screening.

### Screening interview

The standardised screening interview for WISDOM is described elsewhere [1]. Briefly, during the one-hour standardised screening interview the trial nurse explained the currently known harms and benefits of long-term HT, the purpose and procedures of WISDOM, and conducted an extensive eligibility check. If the woman was eligible and willing to participate, informed written consent was sought from the woman and her GP, and an appointment was arranged 2 weeks later with the trial nurse to initiate the 12 week 'run-in' phase prior to randomisation. Details of those randomised and the outcomes of WISDOM are described elsewhere [4].

The number of seminar attendees and non-attendees who completed a screening visit, run-in and randomisation visit was recorded, and a Chi-square test was performed using SPSS 13.0 statistical software to test for differences in recruitment rates between the two groups.

### Feedback questionnaires

In November 2002 (5 weeks after stopping WISDOM), anonymous feedback questionnaires were posted to all 840 women who had completed at least a screening visit for the Adelaide WISDOM recruitment centre [5]. Women were asked whether or not they had attended an initial seminar for WISDOM, and asked to rate on a Likert scale how helpful the seminar, one-to-one visit with the nurse (screening interview) and information sheet were in their decision to participate in WISDOM.

## Results

### Recruitment of women who attended the seminar

**Figure 1:**
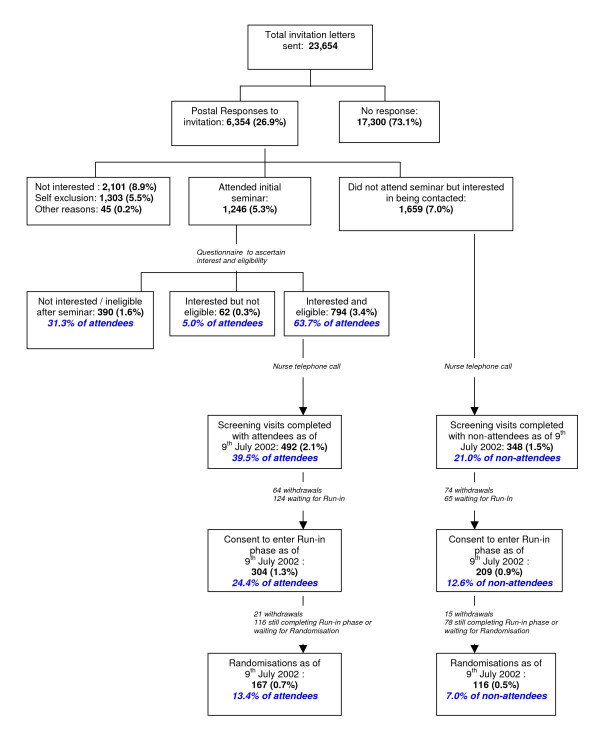
Flowchart of Adelaide recruitment procedure.

Figure 1 illustrates the Adelaide recruitment procedure for WISDOM and the number of seminar attendees and non-attendees who reached the screening, run-in and randomisation phases of the trial. The mean number of invited women attending seminars ranged from 12 – 81 per session (mean 44). This was limited by the size of the practice, and its number of female patients aged 50–69 who received the initial invitation letter. Of the 1246 women who attended a seminar, 492 (39.5%) attended a screening visit with a trial nurse, and 304 (24.4%) and 167 (13.4%) reached the run-in and randomisation phases, respectively. In comparison, of the 1659 women who did not attend a seminar, 348 (21.0%) attended a screening visit, and 209 (12.6%) and 116 (7.0%) reached the run-in and randomisation phases, respectively. Thus, seminar attendees were nearly twice as likely to attend an initial screening visit with a trial nurse (Chi square = 118.6, 1 df, p < 0.001) and go on to the run-in (Chi square = 68.1, 1 df, p < 0.001) and randomisation (Chi square = 33.3, 1 df, p < 0.001) phases of the trial.

A higher proportion of non-attendees (74/348, 21.2%) either withdrew or were not eligible after screening than attendees (64/492, 13.0%)

### Helpfulness of seminar

A total of 618/840 (73.6%) Adelaide participants returned completed feedback questionnaires. A description of the respondents is provided elsewhere [5]. Three hundred and seventy eight (61.2%) of the respondents stated that they had attended an initial seminar prior to enrolment in WISDOM. As shown in Table 1 below, of the 378 women who attended the seminar, 88.9% regarded the seminar as either moderately helpful or very helpful in their decision to participate in WISDOM. One-to-one contact with the trial nurse and written information was regarded as moderately or very helpful by 81.5% and 73.0%, respectively. For women who did not attend the seminar (n = 235), one-to-one contact with the trial nurse and written information was rated as moderately helpful or very helpful by 78.3% and 73.7%, respectively.

**Table 1:** Questionnaire responses to "What was most helpful in your decision to enter WISDOM?"

	Attended seminar		Did not attend seminar	
	N = 378	%	N = 235	%
Reading the information that was sent to you				
Not helpful	3	0.8	0	0
Somewhat helpful	55	14.6	22	9.4
Moderately helpful	142	37.6	77	32.8
Very helpful	134	35.4	96	40.9
Not applicable	18	4.8	8	3.4
Not specified	26	6.9	32	13.6
Attending the information evening				
Not helpful	2	0.5		
Somewhat helpful	11	2.9		
Moderately helpful	77	20.4		
Very helpful	259	68.5		
Not applicable	13	3.4	155	66.0
Not specified	16	4.2	80	34.0
Seeing the trial nurse for the first time				
Not helpful	9	2.4	5	2.1
Somewhat helpful	24	6.3	9	3.8
Moderately helpful	65	17.2	34	14.5
Very helpful	243	64.3	150	63.8
Not applicable	19	5.0	8	3.4
Not specified	18	4.8	29	12.3

### Contact time spent with attendees and non-attendees

**Figure 2:**
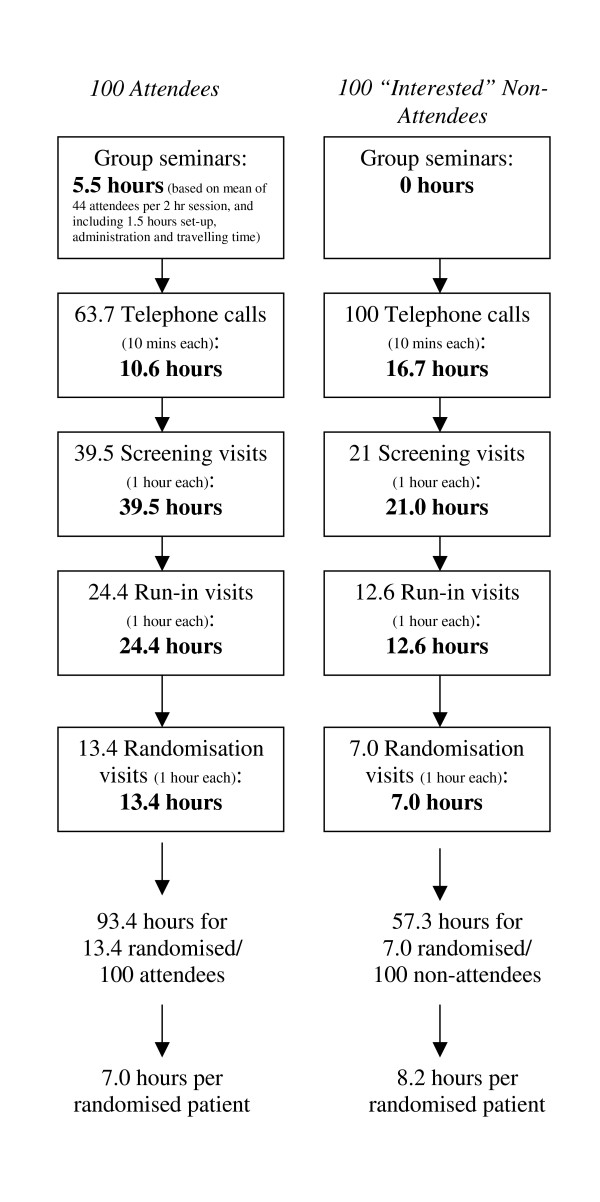
Estimated total contact time required for each group and number of potential participants reaching randomisation (based on 100 potential participants in each group).

As shown in Figure 2, based on a sample of 100 seminar attendees and 100 interested non-attendees, an estimated average of 7.0 hours contact time was required to randomise seminar attendees, compared to 8.2 hours contact time for interested women who did not attend. Further, the number of initial telephone calls required to confirm interest or screen-out uninterested women was lower in the attendee group (63 calls) than in the non-attendee group (100 calls). Since duration of telephone calls was not recorded, we have estimated each call to be 10 mins duration in both groups. However, it is important to note that the purpose of telephone calls to attendees was only to arrange an appointment time for screening and not to discuss the aims and procedures of WISDOM, or to confirm eligibility, so telephone calls to these women were likely to be shorter.

## Discussion

The results of this analysis suggests that running group information seminars to facilitate the recruitment of women for a large randomised placebo controlled trial results in greater numbers screened and randomised, fewer drop-outs, and less total recruitment time spent by the research team. This technique was adopted when it became apparent from the experience of the UK arm of WISDOM that recruitment was slower than had been anticipated from an initial pilot study.

Women who attended a group seminar conducted by our research team were twice as likely to attend an initial screening visit with a trial nurse and enrol to participate than women who did not attend a seminar but only received one-to-one interaction with the trial nurse. It should be emphasised that this study was not a randomised controlled trial to test seminars as an intervention to improve recruitment, but rather, a cross sectional study suggesting that seminars may be a worthwhile recruitment strategy, which could be tested with a stronger research methodology in future. At the very least, this study implies that offering seminars is a good way of differentiating those who will eventually participate in a trial from those who do not.

We believe our recruitment procedure, where women attended an information seminar and screened themselves for eligibility prior to attending a formal screening session with a trial nurse, was more efficient than the procedures used to recruit patients in the UK. Following an extensive notesearch of women patients aged 50–69 years in general practices across the UK, 155,204 women were invited to screening [4]. Although a high number of these women (56,583) agreed to attend screening, only 16% (8980) and 10% (5692) of those screened went on to participate in the run-in and randomisation phases of the trial, respectively [4]. In Adelaide, the notesearching step was omitted in favour of women attending an information seminar and screening themselves for eligibility, and of the 840 women who attended screening, 61% and 34% continued on to participate in the run-in and randomisation phases of the trial.

It is possible that the seminar increased women's motivation to participate. However, as women were not randomly allocated to attend the seminar or receive only one-to-one contact with the trial nurse, the higher participation rate observed amongst seminar attendees may simply be due to selection bias. That is, attendees may have been more motivated than non-attendees to participate in WISDOM upon first receiving the invitation letter, and thus chose to attend the seminar because they wanted to be as informed as possible about the trial before enrolling. It would have been useful to compare the age and sociodemographic characteristics of women in the attendee and non-attendee groups but this information was not collected on reply slips.

Conducting seminars with potential participants prior to enrolment in an RCT may confer several advantages for the participant, research team and general practice. For the participant, a seminar conducted by the research investigator provides expert, up-to-date information about the research area and clinical trial, and a more comprehensive discussion of the characteristics of RCTs, including clinical equipoise, the need for randomisation to placebo or treatment and the need for blinding to treatment. The seminar also gives participants an opportunity to ask questions of senior researchers and to meet and share these questions and concerns with other potential participants, partners, friends and relatives. This group setting may provide participants with the time and space they need to independently consider all facets of the trial before making a personal commitment to participate, and it could be perceived as less intimidating than individual meetings with their GP or trial nurse. The group seminar also confers benefits for the research team. Potential participants, regardless of whether they attended the seminar or not, were telephoned by the trial nurse to discuss participation and invited to a screening visit. However, seminar attendees who recognised that they were ineligible or not interested in participating at the end of the seminar had the opportunity to inform the research team via the post-seminar questionnaire, and so these women did not need to be telephoned. Further, the telephone calls to attendees were probably shorter, since there was no need to spend time explaining the purpose, procedures or eligibility criteria of the trial. In addition, seminar attendees were less inclined to drop-out after their screening visit than non-attendees.

Although numerous studies have evaluated the effectiveness of various recruitment strategies, to our knowledge few have focused on the effectiveness of group seminars for increasing recruitment. One research team found group information sessions to be a more efficient and cost effective recruitment strategy than individual sessions when recruiting female university students for a community-based trial of chlamydia screening to prevent pelvic inflammatory disease [6]. Another team found group meetings with families to be a useful strategy for recruiting children to RCTs, where parents must provide consent [7]. In contrast, an educational intervention that included group seminars was not more effective than written information for increasing recruitment of older cancer patients to treatment trials [8]. These studies suggest that, if seminars are indeed useful, their effectiveness may vary in different populations.

Our recruitment process enables research teams to take the responsibility and effort of recruitment away from busy GPs and practice staff. There was no need for GPs or practice nurses to screen their patient records to identify potentially eligible patients, since all women within the eligible age range (50–69 years) were sent detailed trial information, and asked to screen themselves for initial eligibility at the invitation letter stage, at the seminar (via post-seminar questionnaire) or during their telephone call with the trial nurse. Secondly, there was no need for GPs or practice nurses to recruit participants since all seminars were conducted by the research team (BJP, NPS and AHM), and telephone calls and screening interviews (as well as all subsequent trial visits) were conducted by dedicated trial nurses. GPs were only required to assist the trial nurses after enrolment, when monitoring participant health outcomes and reporting adverse events was required.

The proportion of respondents to our initial invitation letter was considerably low, at 27%. However, this is perhaps not surprising given that we asked women to screen themselves for eligibility using an extensive list of inclusion and exclusion criteria provided in the information sheet. One of the exclusions was current use of HT for women with a uterus and a range of health conditions including breast cancer, any other cancer in the last ten years, endometriosis, venous thromboembolism, gall bladder disease, heart disease and stroke. According to a 2002 South Australian Health Omnibus survey, 28% of women aged over 50 were currently using HT in 2000, with ever-use of HT at 43% [9]. Thus, many women who were sent our invitation letter would have been using HT or had experienced one of the health exclusion criteria, so believed themselves to be ineligible and may have disregarded the letter without responding. Many other women who had taken HT previously may have experienced side effects and so may not have been willing to enter a trial where they may required to take HT again.

## Conclusion

Group seminars conducted by the research team may be a useful strategy for maximising participant recruitment to RCTs, particularly in the busy general practice setting. Seminars may increase a participant's understanding of the research topic and the principles of RCTs, allowing them to make a more informed choice about participation and their eligibility. They allow potential trial participants to meet the principal investigators, other potential participants and hear each other's views in a relaxed setting. It is possible that motivation to join a long-term study, and any altruistic feelings for helping medical research, is enhanced by hearing directly from senior investigators and by hearing the questions and reactions of other women in the audience. Providing information about the trial's background, purpose and procedures to a group rather than individually, and allowing participants to screen themselves for eligibility, may significantly reduce the amount of time spent by research or practice staff recruiting participants. Nevertheless, given the cross-sectional nature of our study, the potential for selection bias was a possibility, and thus future randomised controlled trials to test seminars as an intervention to improve recruitment are needed.

## Competing interests

The author(s) declare that they have no competing interests.

## Authors' contributions

BJP was the Trial Coordinator for the Australian arm of WISDOM, and drafted the manuscript

NPS was the Medical Director for the Australian arm of WISDOM, recruited general practitioners and conducted group seminars with participants.

AHM was Principle Investigator for the Australian arm of WISDOM, conceived of the Australian recruitment procedure and conducted group seminars with participants
